# Prevalence of malnutrition among pre-school children in, South-east Nigeria

**DOI:** 10.1186/s13052-014-0075-5

**Published:** 2014-09-11

**Authors:** Pius C Manyike, Josephat M Chinawa, Agozie Ubesie, Herbert A Obu, Odutola I Odetunde, Awoere T Chinawa

**Affiliations:** Federal Teaching Hospital Abakiliki, Abakiliki, Nigeria; Department of pediatrics, University of Nigeria/University of Nigeria Teaching Hospital (UNTH), Ituku- Ozalla, Enugu State Nigeria; Department of Community Medicine, University of Nigeria/University of Nigeria Teaching Hospital (UNTH), Ituku- Ozalla, Enugu State Nigeria

**Keywords:** Z-score, Malnutrition, Pre-school, Children, Nigeria

## Abstract

**Background:**

Malnutrition can be defined as a state of nutrition where the weight for age, height for age and weight for height indices are below -2 Z-score of the NCHS reference. It has posed a great economic burden to the developing world.

**Objectives:**

The objective of this study is to assess the prevalence of malnutrition among pre-school children in abakiliki in Ebonyi state of Nigeria.

**Methods:**

This is a cross-sectional studies that assess the prevalence of malnutrition and associated factors among children aged 1-5 years attending nursery and primary schools. Nutritional assessment was done using anthropometry and clinical examination.

**Results:**

A total of 616 children aged one to 5 years were enrolled into this study. Three hundred and sixty-seven (59.6%) were males while 249 (40.4%) were females. Sixty of the 616 children (9.7%) had acute malnutrition based on WHZ-score. Moderate acute malnutrition (MAM) was present in 33 children (5.3%) while 27 (4.4%) had severe acute malnutrition.

**Conclusions:**

The prevalence of global and severe acute malnutrition using z-score is 9.7% and 4.4% respectively while that of stunting is 9.9% with a male preponderance.

## Introduction

Malnutrition is an unbearable burden not only on the health systems, but the entire socio-cultural and economic status of the society [1]. Malnutrition can be defined as a state of nutrition where the weight for age, height for age and weight for height indices are below -2 Z-score of the NCHS reference [2]. It constitutes a major public health problem in developing world and serves as the most important risk factor for the burden of disease especially among preschool children. Though the United Nations has also adopted the Millennium Development goals seek to halve childhood malnutrition indicators by 2015, yet malnutrition still contributes significant morbidity and mortality among preschool children [3]. For instance about 5 million children, especially those under five, died worldwide directly or indirectly due to malnutrition and 9 children/minute die as a result of malnutrition. In this regard, World Health Organization has identified childhood malnutrition as the most lethal form of malnutrition [4,5]. Globally, it is estimated that there are nearly 20 million children who are severely acutely malnourished, most of them live in south Asia and in sub-Saharan Africa [6].

The worldwide malnutrition estimation rates indicate that 35.8% of preschool children in developing countries are underweight, 42.7% are stunted, and 9.2% are wasted [7]. In children aged 6–59 months, an arm circumference less than 110 mm is also indicative of severe acute malnutrition. Apart from marasmus and kwashiorkor (the 2 forms of protein- energy malnutrition) micronutrient deficiencies also exist among these children. Deficiencies in iron, iodine, vitamin A and zinc are the most common in developing countries. In these communities, a high prevalence of poor diet and infectious disease regularly unites into a vicious cycle [8]. There have been several attempts made at classifying degrees of malnutrition using Wellcome classification, Weight-for-age classifications by Gomez and I.A.P and Height-for-age and Weight-for-height classifications by Waterlow’s [8]. The Wellcome classification is frequently used and Waterlow’s less frequently [9]. These classifications use different sets of reference data and each system employ different cut-off points to decide who is normal and who falls under mild, moderate, or severe undernutrition [9]. The cut-off points however are usually a certain percentage of the mean/median or a percentile, of the reference population. Unfortunately, most of the cut-off points are admittedly arbitrary and do not carry a prognostic significance for any given individual child [2].

None of these classifications address all the three indices of undernutrition - Stunting, Wasting and Underweight. Stunting (Low height-for-Age) is an indicator of chronic undernutrition due to prolonged food deprivation and/or illness; Wasting (Low weight for height) is an indicator of acute undernutrition, the result of more recent food deprivation and/or illness; Underweight (Low weight-for-age) is used as a composite measure to reflect both acute and chronic undernutrition [2].

Currently, the WHO recommended the use Z-Score or SD system to grade undernutrition. This method measures all the three indices and expresses the results in terms of Z scores or standard deviation units. Children who are more than 2 SD below the reference median (i.e. a Z-Score of less than -2) are considered to be undernourished i.e. to be stunted, wasted or to be underweight. Children with measurements below 3 SD (a Z-Score of less than-3) are considered to be severely undernourished [2].

Evaluation of prevalence of malnutrition among pre-school children using z-score is a very vital issue often under reported in pediatrics practice and in this part of the world, its importance therefore cannot be downplayed especially its impact on health which had been mentioned above.

This study therefore is aimed at determining the prevalence and pattern of malnutrition among pre-school children using z-score. The results will help us to know the burden of this disease. This will enable us to establish a baseline data where other related issue will hinge on.

We are not aware of any study of this nature from this environment. It is hoped that this will add to the body of knowledge available on these disorders and the findings of this study could form the template for intervention strategies in helping reduce this social malaise and managing such cases.

## Methods

### Study design

This is a cross-sectional studies that assess the prevalence of malnutrition and associated factors among children aged 1-5 years.

### Study area and period

The study was conducted from selected two nursery and two primary schools in Abakiliki, Ebonyi state. Abakiliki is the capital of Ebonyi state, south-east Nigeria and has a population of 134102 according to the GeoNames geographical database [10].

### Study population and study procedure

Source populations were all children aged 1-5 years attending nursery and primary schools in Abakiliki. Written Consent and approval were given by the school authorities where this study was carried out while verbal consent was obtained from parents of the pupils.

Confidentiality of responses was also conveyed. Pupils were chosen by systemic sampling from nursery one to primary one. The study population are made up of people from mainly middle and lower class. Nutritional assessment was done using anthropometry. Children were weighed and measured as per the WHO guidelines on Anthropometry [11,12].

Stadiometer (Floor type model with sensitivity of 0.1 cm and 0.1 kg): was used to measure the height and weight of the children. It typically consists of a vertical ruler with a sliding horizontal rod or paddle which is adjusted to rest on top of the head. The mid arm circumference was measure with a measuring tape on two consecutive times. The sensitivity is 0.1 cm.

Children aged 1-5 years who live and attend schools in nursery and primary schools in Abakiliki whose teachers gave consent were included in this study while children who were seriously ill and those whose parents did not give consents were excluded.

The objective of this study is to assess the prevalence of malnutrition among children aged 1-5 years attending nursery and primary schools in Abakiliki in Ebonyi state of Nigeria.

#### Diagnostic methods

Moderate acute malnutrition was defined as weight for height of ≥ -3 and < -2 z-score and mid arm circumference (MAC) of 11 - <12.5 cm. Severe acute malnutrition was defined as weight for height z-score of < -3 and MAC of <11 cm. Stunting was defined as height for age z-score of < -2.

### Data analysis

Data analysis was with Statistical Package for Social Sciences (SPSS) version 19 (Chicago IL). Chi-square test was used to test for significant association of the proportion. A p-value of < 0.5 was regarded as significant. All reported p-values were 2-sided.

## Result

### Demography

A total of 616 children aged one to 5 years were enrolled into this study. Three hundred and sixty-seven (59.6%) were males while 249 (40.4%) were females. The distribution of the children according to age and gender is shown in Table 1.

**Table 1:** **Distribution of the children according to age and gender**

**Gender**	**Male (%)**	**Female (%)**
**Age (years)**		
1	74 (20.2)	47 (18.9)
2	106 (28.9)	74 (29.7)
3	112 (30.5)	59 (23.7)
4	56 (15.3)	51 (20.5)
5	19 (5.2)	18 (7.2)
**Total**	**367 (100.0)**	**249 (100.0)**

### Acute malnutrition

Sixty of the 616 children (9.7%) had acute malnutrition based on WHZ-score. Moderate acute malnutrition (MAM) was present in 33 children (5.4%) while 27 (4.4%) had severe acute malnutrition. However, MAM and SAM based on MAC criteria was present in only 10 (1.6%) and 1 (0.2%) of the 616 children respectively. Thirty-seven of the 330 males (10.1%) compared to 23 of the 226 females (9.2%) had acute malnutrition (p = 0.78). Prevalence of acute malnutrition was highest among the one (12.4%) and two year olds (12.8%) and least among the four year old (4.7%) (p = 0.14). A higher proportion of the males (6%) than females (4.4%) had MAM. Conversely, a slightly higher proportion of the females (4.8%) than males (4.1%) had SAM. Nine of the 47 one year old females (19.1%) compared to 6 of 74 males (8.1%) had acute malnutrition. This difference showed a trend that failed to attain significance (p = 0.09). Conversely, 18 of 106 two- year old males (17.0%) compared to 5 of 74 females of same age had acute malnutrition. This difference also showed a trend that failed to attain statistical significance (p = 0.07). Detailed comparison of acute malnutrition between the genders according to their ages is shown in Table 2.

**Table 2:** **Acute malnutrition between the genders according to their ages**

**Gender**	**Male (%)**	**Female (%)**	**p-value**
**Age (years)**			
1	6 (8.1)	9 (19.1)	0.09
2	18 (17.0)	5 (6.8)	0.07
3	8 (7.1)	5 (8.5)	0.77
4	3 (5.4)	2 (3.9)	1.0
5	2 (10.5)	2 (11.1)	0.78

### Stunting

Sixty-one of the 616 children (9.9%) were stunted. Thirty nine of the 367 males (10.6%) were stunted compared to 22 of the 249 females (8.8%) although this difference was not statistically significant (p = 0.47). Across the age groups, a higher proportion of the two (19.4%) and three year olds (10.5%) compared to one (3.3%), four (2.8%) and five (2.7%) year olds were stunted (p < 0.001). However, 23 of 106 two-year old males compared to 12 of 74 females of same age were stunted (p = 0.45). Additionally, 13 of 112 males (11.6%) compared to 5 of 59 females aged 3 years were stunted (p = 0.61). Detailed comparison of stunting between the genders according to their ages is shown in Table 3.

**Table 3:** **Comparison of prevalence of stunting between the genders according to their ages**

**Gender**	**Male (%)**	**Female (%)**	**p-value**
**Age (years)**			
1	2 (2.7)	2 (4.3)	0.64
2	23 (21.7)	12 (16.2)	0.45
3	5 (8.5)	13 (11.6)	0.61
4	3 (5.9)	0 (0.0)	0.11
5	0 (0.0)	1 (5.3)	1

## Discussion

The results of this study show acute malnutrition in this metropolis do exit, though there is lack of cultural and social recognition of this hidden malaise. The prevalence of stunting from our study is 9.9%. The result of this study revealed that, the prevalence of stunting is low when compared with a community cross-sectional study conducted in rural kebeles of Haramaya and NorthShewa district all in Ethiopia where a prevalence of 32.4% and 47.7% respectively were obtained [13,14]. The variations of prevalence could be due to geographical and racial differences. The large sample size used in this study could also be contributory. Stunting is slightly higher in males when compared to females, though not statistically significant. Henry and colleagues [15] working in ten sub-Saharan countries also identified a slight male preponderance.

Possible reasons for the observed sex differences in these studies mainly centres on behavioural patterns [16]. For instance, Svedberg et al. [17] noted a slight anthropometric advantage shown by females in many countries and suggest a historical pattern of preferential treatment of females due to the high value placed on women’s agricultural labour. Cronk [18] and colleagues in Kenya also suggested that favouritism towards daughters occurred as a result of lowered socio-economic status. However, there are also studies that report greater social valorization of sons at the detriment of daughters [19], including dietary discrimination [20], thereby dispelling conclusions of a nutritionally advantaged position of female over male children. Other reasons could be that boys were more influenced by environmental stress than girls [20,21]. There were significant age differences in stunting in that a higher proportion of the two and three year olds compared to one, four and five year olds were stunted. This finding is in keeping with the studies of Beatrice et al. [22] and an Indian study [23] who found the highest prevalence of stunting in children aged 36-47 months. We attribute this similar findings to poor weaning and complementary feeding practices, which contribute to inadequate energy and protein intake [24]. However, we did not collect data to determine the impact of feeding practices on nutritional status. Other reasons could be that in the second year of life, with introduction to the family diet, children become more responsible for feeding themselves but often do not have access to adequate amounts of solid food [25].

We noted from this study that the prevalence of severe acute malnutrition and global malnutrition is 4.4% and 9.7% respectively. This is at variance with the global prevalence of 16.1 obtained by Casie et al. [16] in Chad and that obtained in Tanzania by Sunguya et al. [21] who obtained a global prevalence of 13.6%. The low prevalence obtained in our study when compared to Chad and Tanzania study is because the latter was a retrospective hospital study, which included all malnourished in-patients admitted over a year period. Moreso z-score was not used to classify acute malnutrition in Tanzania study.

The prevalence of moderate acute malnutrition was 5.4% with a slight male preponderance. This is lower than the 16% reported by WHO with a female preponderance [26]. A study that cuts across several countries with a very large sample size could explain this difference in prevalence. It is interesting to note that the prevalence of acute malnutrition was highest among the one and two year olds but least among the four year old. This was in tandem to the findings of Beatrice et al. [22] who noted Severe wasting was most common among children aged 24-35 months. The reason for this prevalence is as explained above [17-21]. Severe wasting is commoner among females than males though this is not significant. This finding is in keeping with other studies [17-21].

Using MAC alone, a prevalence of severe malnutrition of 1.6% was obtained in this study. This is very low when compared with the 9% obtained by WHO [26]. Although, the WHO study work involved several countries, it should be noted that MUAC is not an ideal tool in monitoring acute malnutrition in children. This is due to the fact that with a new WHO curve, the performance of MUAC measurements, in terms of sensitivity and specificity, was very low [26]. In rural communities, MUAC could be a valuable tool for use by CHWs for early detection of acute malnutrition in infants [25]. However, reliability of MUAC measurement in early infancy is unknown, and cut-off values to determine intervention thresholds have not been defined [25].

## Conclusions

The prevalence of global and severe acute malnutrition using z-score is 9.7% and 4.4% respectively while that of stunting is 9.9% with a male preponderance.

## Recommendation

Study of the prevalence of malnutrition in a larger community setting will make the impact and attendant problems to be appreciated better.
